# Precise Ultrasound Neuromodulation in a Deep Brain Region Using Nano Gas Vesicles as Actuators

**DOI:** 10.1002/advs.202101934

**Published:** 2021-09-21

**Authors:** Xuandi Hou, Zhihai Qiu, Quanxiang Xian, Shashwati Kala, Jianing Jing, Kin Fung Wong, Jiejun Zhu, Jinghui Guo, Ting Zhu, Minyi Yang, Lei Sun

**Affiliations:** ^1^ Department of Biomedical Engineering The Hong Kong Polytechnic University Hung Hom Hong Kong SAR 999077 P. R. China

**Keywords:** low‐frequency ultrasound, nano gas vesicles, transcranial ultrasound stimulation, ultrasonic neuromodulation

## Abstract

Ultrasound is a promising new modality for non‐invasive neuromodulation. Applied transcranially, it can be focused down to the millimeter or centimeter range. The ability to improve the treatment's spatial resolution to a targeted brain region could help to improve its effectiveness, depending upon the application. The present paper details a neurostimulation scheme using gas‐filled nanostructures, gas vesicles (GVs), as actuators for improving the efficacy and precision of ultrasound stimuli. Sonicated primary neurons display dose‐dependent, repeatable Ca^2+^ responses, closely synced to stimuli, and increased nuclear expression of the activation marker c‐Fos in the presence of GVs. GV‐mediated ultrasound triggered rapid and reversible Ca^2+^ responses in vivo and could selectively evoke neuronal activation in a deep‐seated brain region. Further investigation indicate that mechanosensitive ion channels are important mediators of this effect. GVs themselves and the treatment scheme are also found not to induce significant cytotoxicity, apoptosis, or membrane poration in treated cells. Altogether, this study demonstrates a simple and effective method to achieve enhanced and better‐targeted neurostimulation with non‐invasive low‐intensity ultrasound.

## Graphical abstract

This study utilizes ultrasound‐responsive nanoscale gas vesicles (GVs) to mediate controllable transcranial ultrasound stimulation. Sonicated neurons and mouse brain display reversible and repeatable Ca^2+^ responses, and increased c‐Fos expression in the presence of GVs. Mechanosensitive ion channels are found to be involved in mediating the stimulation. This strategy enables targeted stimulation in a deep brain region with high spatiotemporal resolution.

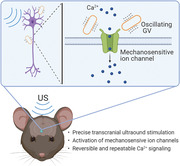

## 1 Introduction

Neurostimulation techniques have expanded greatly over the past several years, and have been used to probe neural systems and to treat neurological disorders. Among the more prominent modalities being developed for this purpose is ultrasound (US). Ultrasound is a form of mechanical energy whose ability to non‐invasively pass through the skull to deep regions of the brain has spurred many to try and apply it for neuromodulation.^[^ ^1^, ^2^ ^]^ Experiments in many different animal species have shown successful stimulation of various brain regions in rodents,^[^ ^3^ ^]^ rabbits,^[^ ^4^ ^]^ pigs,^[^ ^5^ ^]^ sheep,^[^ ^6^ ^]^ and non‐human primates.^[^ ^7^ ^]^ Low‐intensity ultrasound has been used to stimulate various brain regions of the human, including the thalamus,^[^ ^8^ ^]^ the prefrontal, visual,^[^ ^9^ ^]^ motor,^[^ ^10^ ^]^ and somatosensory cortices.^[^ ^11^, ^12^, ^13^, ^14^ ^]^ It is also under study as a possible treatment for a range of neurological disorders, such as Alzheimer's disease,^[^ ^15^, ^16^ ^]^ Parkinson's disease,^[^ ^17^, ^18^, ^19^ ^]^ epilepsy,^[^ ^20^ ^]^ depression,^[^ ^21^ ^]^ and amyotrophic lateral sclerosis.^[^ ^22^ ^]^ Ultrasound has thus shown the ability to affect the functioning of the central nervous system without significant accompanying damage.

One of ultrasound's primary attributes is that it can be delivered with good spatial resolution to deep regions of the brain without surgical invasion. However, given the relatively low frequencies of ultrasound required to successfully pass through an intact skull, the corresponding diffraction‐limited spatial resolution would be in the millimeter to centimeter range.^[^ ^23^ ^]^ Moreover, the heterogeneity and unpredictable acoustic properties of different skulls could significantly distort the ultrasound's focus. Another complication is that the brain is understood to have some inherent level of sensitivity to ultrasound which is spread unevenly across various regions, through mechanisms like mechanosensitive ion channels,^[^ ^24^ ^]^ cytoskeleton and cell‐ECM interactions.^[^ ^25^ ^]^ This raises the possibility that some regions of the brain may have low mechanosensitivity, possibly reducing the potency of ultrasound stimulation. Thus, it would be helpful if some specific brain region or cells within a region could be preferentially sensitized to mechanical stimulation, to enable more efficient neuromodulation.

One way to achieve this aim is through the use of nanoparticles as localized force actuators. Noteworthy approaches that have been demonstrated include, gold nanoparticle‐assisted photothermal stimulation,^[^ ^26^ ^]^ upconversion nanoparticle‐mediated near‐infrared optogenetics,^[^ ^27^ ^]^ and magnetic nanoparticle‐based magnetothermal/magnetomechanical stimulation.^[^ ^28^, ^29^, ^30^ ^]^ In these instances, nanoparticles have been used as mediators to successfully improve the range and targetability of various techniques, or to decrease the invasiveness of the treatment's scheme. A candidate for such an ultrasound actuator is nano‐sized protein structures extracted from cyanobacteria, called gas vesicles (GVs). GVs are hollow protein shells with an impressive ability to enhance ultrasound contrast, due to non‐linear signals generated by ultrasound‐driven buckling effects, making them a unique type of nanosized ultrasound contrast agent.^[^ ^31^ ^]^ Such acoustic oscillations can also transmit mechanical perturbations to the surrounding environment in a manner similar to microbubbles (MBs). The presence of GVs could thus lower the threshold for ultrasound to successfully stimulate neurons by mediating the localized transmission of mechanical energy. This could enable the application of low‐intensity, low‐frequency ultrasound (LILFU) for direct stimulation of the brain. Further, since only neurons with GVs nearby would be stimulated by LILFU, this could allow for increased spatial resolution for ultrasound stimulation even in circumstances where the focal spot may not be easily controllable. Thus, we hypothesized that GVs oscillating in a low‐intensity ultrasound field could serve as actuators enabling the stimulation of proximate neurons.

In the present study we demonstrate a GV‐actuated strategy to achieve controllable ultrasound neuronal stimulation in a targeted brain region. In the presence of GVs, we were able to stimulate primary neurons with low intensity ultrasound to induce Ca^2+^ influx and neuronal activation, but not otherwise. The neuronal responses were dose‐dependent and reversible, as well as closely temporally‐tied to the US stimuli. We found that the calcium dynamics were not significantly attributable to harmful poration in cell membranes, and mechanosensitive cation channels were significantly involved in mediating the stimulation. We were also able to selectively stimulate calcium responses and neuronal activation in a deep‐seated region of the mouse brain, the ventral tegmental area (VTA), using this GVs+US scheme. Taken together, we provide evidence for a simple and efficient method of precise ultrasound neuromodulation by using a single element plane transducer, in vivo and in vitro, through the use of non‐toxic GVs as actuators.

## 2 Results

### 2.1 Characterization of GVs’ Properties

**Figure 1:**
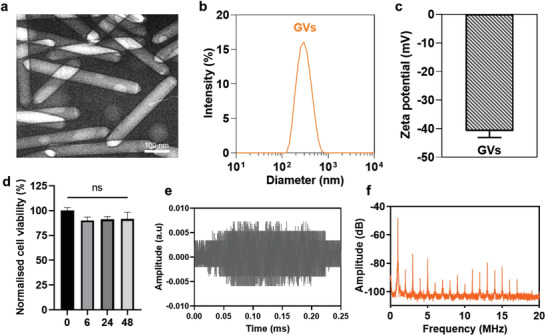
Basic characterization of the prepared GVs. a) Transmission electron microscopy (TEM) image of the prepared GVs. Scale bar represents 100 nm. b) Number‐averaged diameter of GVs in deionized (DI) H_2_O as measured by dynamic light scattering (DLS). Data represent the mean of three independent experiments. c) Zeta potential of GVs in DI H_2_O. Bar represents mean ± SD of three independent experiments. d) Cytotoxicity of GVs (0.8 nm), as measured by an MTT test. Primary neurons were exposed to GVs in medium for the stated amounts of time. Bars represent the mean ± SEM of three independent experiments. No significant differences were found by one‐way ANOVA. e) Representative time‐domain waveform of backscattered signals from a purified GVs suspension (0.8 nm) sonicated by a 1.0 MHz tone burst sinusoidal wave at 0.28 MPa peak negative pressure, after one burst interval (300 cycles). f) Averaged frequency spectrum of backscattered signals from purified GVs suspension under the same sonicating conditions as in (e).

GVs were prepared from *Anabaena flos‐aquae* through tonic cell lysis and centrifugally‐assisted flotation.^[^ ^32^, ^33^ ^]^ They were found to typically be 50–100 nm in width and 100–500 nm long (**Figure** **1**a,b). The zeta potential of the GVs was −40 ± 5 mV, indicating a suitable surface charge for colloidal stability (Figure 1c).^[^ ^34^ ^]^ We also found that our prepared GVs were not cytotoxic on their own to primary neurons in culture (Figure 1d). Primary neurons were also found not to internalize GVs labeled with fluorescein when incubated with them for up to 48 h, and no obvious change were seen in neuronal morphology (Figure S1a, Supporting Information). The GVs remained well‐dispersed in PBS for up to 48 h, observable as a milky, near‐opaque solution, and they separated into a floating layer after 3 weeks (Figure S1b, Supporting Information). In the present study, all US experiments were performed within 1 h of adding GVs to cells, so the GVs’ inherent buoyancy would likely not have been a hindrance. In all, our prepared GVs were found to be nano‐sized, stable and non‐cytotoxic.

US is known to induce both stable and inertial cavitation.^[^ ^35^ ^]^ Generally, stable cavitation occurs at relatively low ultrasound intensities, caused by size changes of gas‐filled bubbles in a sustained, periodic manner. Inertial cavitation usually occurs at high ultrasound intensities, when gas bubbles collapse, generating a shock wave that could cause significant cell damage. We wanted to control ultrasound intensity such that it would enable the GVs to generate robust stable cavitation but not inertial cavitation, which required characterizing the GVs’ responses in an ultrasound field. Hence, we performed passive cavitation detection, exposing GVs suspensions to 0.28 MPa peak negative pressure pulsed US (Figure S1c,d, Supporting Information). We observed the backscattered signals in the time‐ and frequency‐domains to monitor the patterns of cavitation produced. We found no broadband signal and only the appearance of first to 17th harmonic signals (Figure 1e,f), indicating that no inertial cavitation occurred when the GVs were sonicated in our setup. Crucially, 0.28 MPa was the highest acoustic pressure used in the entire study, making inertial cavitation unlikely at the range of intensities used in the various following experiments. Further, *Anabaena* GVs are known to have a critical collapse pressure, ranging from 0.44 to 0.605 MPa.^[^ ^36^ ^]^ Therefore, GVs were expected to maintain their integrity under low‐intensity US stimulation in our experiments. This is in keeping with the general preference for keeping US intensities low to minimize the chance of thermal effects and cell damage.^[^ ^24^, ^37^, ^38^, ^39^ ^]^

### 2.2 GVs Enable Efficient Neuromodulation by Low‐Intensity Ultrasound

**Figure 2:**
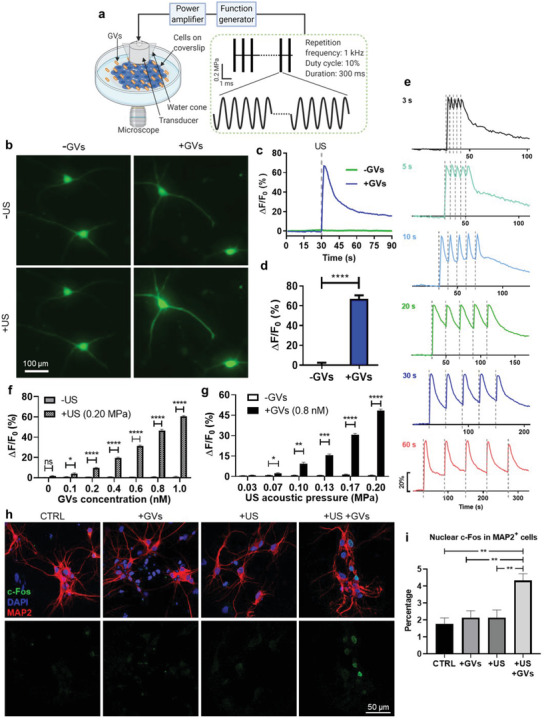
GVs enable low‐intensity ultrasound to stimulate activity in primary neurons. a) Schematic illustration of the GV‐mediated ultrasound setup for recording cells. GVs were mixed into cell culture medium. Cellular response upon US+GVs stimulation was observed in real time. b) Representative images of GCaMP6s fluorescence in primary neurons with or without GVs, before and after 0.20 MPa ultrasound. c) Ca^2+^ imaging time course of neurons in (b). Δ*F*/*F*_0_: the change in fluorescence/initial baseline. d) Ca^2+^ response of neurons to stimulation by 0.20 MPa ultrasound. Bars represent mean ± SD from three independent experiments. ****p < 0.0001, two‐tailed unpaired *t*‐test. e) Time‐resolved Ca^2+^ responses of neurons stimulated by five ultrasound pulses at varying intervals. f) Ca^2+^ response of cells to varying GV concentrations, 0.20 MPa ultrasound. Bars represent mean ± SEM of three independent experiments. *p < 0.05, ****p < 0.0001, two‐tailed unpaired *t*‐test with Holm–Sidak correction. g) Ca^2+^ response of cells to varying ultrasound intensities, 0.8 nm GVs. Bars represent mean ± SEM of three independent experiments. *p < 0.05, **p < 0.01, ***p < 0.001, ****p < 0.0001, two‐tailed unpaired *t*‐test with Holm–Sidak correction. h) Representative IF images of c‐Fos and MAP2 staining in untreated cells (CTRL), and cells treated with the indicated combinations of ultrasound (0.20 MPa, +US) or GVs (0.8 nm, +GVs). i) Quantified results of nuclear c‐Fos staining in MAP2^+^ cells after various treatments, as in (h). Bars represent mean ± SEM from four independent experiments. **p < 0.01, one‐way ANOVA with post‐hoc Tukey test.

We first tested the GVs+US scheme by observing Ca^2+^ responses in rat primary cortical neurons (1.0 MHz center frequency, 0.20 MPa ultrasound and 0.8 nm GVs unless otherwise indicated). Neurons expressing the genetically‐encoded calcium sensor GCaMP6s were imaged during US stimulation. GCaMP6s fluorescence increased quickly and dramatically when stimulated with an ultrasound pulse in the presence of GVs, but not without them, and the fluorescence gradually returned to the baseline without further stimulation (**Figure** **2**b–d). Next, we tested whether neuronal activation could be induced repeatedly. Five 300 ms pulses were delivered to cells at varying intervals in the presence of GVs and the temporal profiles of the cells’ Ca^2+^ response was charted. Stable and reversible calcium transients were seen to quickly follow each pulse, and the neurons were able to recover after each pulse when given enough time (max Δ*F*/*F*_0_ = 46 ± 1.8%, five pulses) (Figure 2e and Video S1, Supporting Information). Aside from primary neurons, we also observed the same pattern of responses, albeit at lower amplitudes, in the mouse hippocampal cell line mHippoE‐18 (referred to as “CLU199” in this manuscript). In the presence of GVs, ultrasound triggered robust, repeatable, and rapid calcium responses from cells after which calcium levels would gradually recover, while no response was seen without GVs (Figure S2a–d, Supporting Information). The cellular response was found to vary with, both the concentration of GVs and the acoustic pressure applied, both in primary neurons (Figure 2f,g) and in CLU199 cells (Figure S2e,f, Supporting Information). US could elicit significant neuronal Ca^2+^ influx in the presence of GVs but not without. GVs+US‐induced Ca^2+^ responses were dose‐dependent both on the concentration of GVs as well as the intensity of ultrasound applied. Δ*F*/*F*_0_ ranged from 3.9 ± 1.3% (0.1 nm GVs) to 60.4 ± 1.6% (1.0 nm GVs) (Figure 2f), and from 2.4 ± 1.1% (0.07 MPa US) to 47.6 ± 2.1% (0.2 MPa US) (Figure 2g). These data help to establish that the responses observed were indeed caused by the GVs+US treatment, and also reveal how such a combination treatment can easily be tweaked to suit the degree of response desired. A GV concentration as low as 0.1 nm was sufficient to induce a significantly higher response than without GVs, showing that GVs could indeed lower the acoustic pressure threshold needed for Ca^2+^ response, which would otherwise require increased ultrasound intensity.^[^ ^2^ ^]^ Finally, when treated with GVs+US primary neurons showed approximately double the nuclear c‐Fos expression, a marker of neuronal activity downstream of Ca^2+^ influx,^[^ ^40^ ^]^ than when they were untreated or exposed to only GVs or ultrasound (Figure 2h,i). Thus, we were able to use GVs to efficiently stimulate activation in primary neurons with short bursts of low‐intensity ultrasound.

### 2.3 Ca^2+^ Influx Induced by GV‐Mediated Ultrasound Stimulation Involves Mechanoresponsive Elements in Cells, Not Obvious Membrane Poration

**Figure 3:**
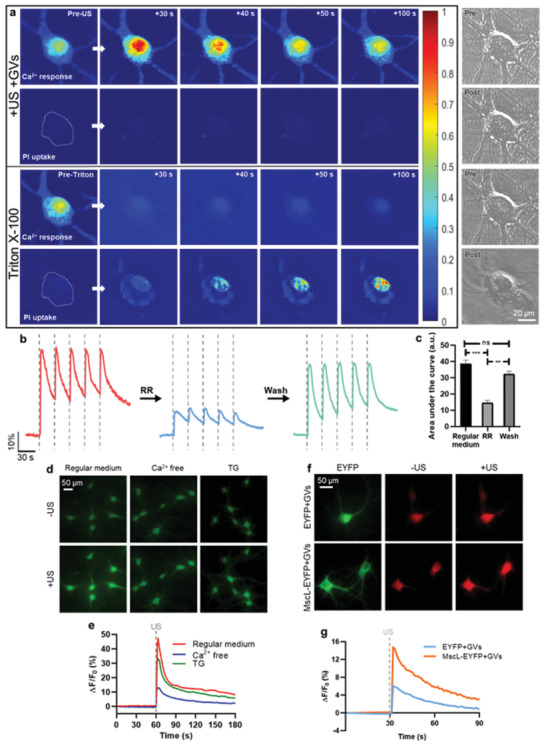
Mechanosensitive ion channels are an important mechanism for GV‐mediated ultrasound stimulation. a) Upper: Calcium response and PI uptake (indicating membrane integrity) during US+GVs stimulation. Lower: Calcium response and PI uptake (indicating membrane integrity) following addition of 0.2 mm Triton X‐100 as a positive control for loss of membrane integrity. Right: Brightfield images of the images cells before and after ultrasound + GVs or Triton X‐100. All images shown in this panel are representative. b) Time‐resolved calcium responses of neurons during US+GVs stimulation, first as normal, then in the presence of mechanosensitive ion channel blocker ruthenium red (RR), and then after RR was washed away. c) Quantification of area under the curve before, during and after RR treatment as shown in (b). Bars represent the mean ± SEM of three independent experiments. **p < 0.01, ***p < 0.001, one‐way ANOVA with post‐hoc Tukey test. d) Representative images of calcium responses of neurons in regular medium, Ca^2+^‐free solution and Thapsigargin (TG). e) Time‐course calcium imaging of cells before and after ultrasound + GVs stimulation in the three solutions indicated in (d). f) Primary cultured cortical neurons expressing MscL‐G22S‐EYFP or EYFP were exposed to a 100 *μ*s pulse of 0.13 MPa ultrasound. Representative images of X‐Rhodamine fluorescence before and after ultrasound, as well as EYFP expression are shown. g) Time‐course imaging of the results shown in (f).

A possible confounding factor in our experiments was that sonoporation, of the kind that is typically induced by ultrasound in the presence of microbubbles, is known to play a role in initiating Ca^2+^ response.^[^ ^41^, ^42^ ^]^ Although only stable cavitation was detected in our system, the acellular evidence cannot address whether our treatment was causing pore formation in cell membranes. Thus, we performed a membrane integrity assay to see whether sonoporation was involved in the Ca^2+^ responses to the GV‐mediated ultrasound treatment. We used the membrane impermeable dye propidium iodide (PI) and observed whether it could penetrate the cell membrane during the stimulation. Insonated primary neurons in the presence of GVs evoked Ca^2+^ influx, but no PI could be detected inside the cells; brightfield imaging also showed that the cells maintained their morphology following the treatment (**Figure** **3**a; Figure S3a and Video S2, Supporting Information). To contrast, Triton X‐100 was used as a positive control for membrane permeation,^[^ ^43^ ^]^ and PI influx was seen within 30 s of its addition and continued to increase for the remainder of the assay, while the intracellular calcium signal decreased and the cell was visibly damaged (Figure 3a and Figure S3a, Supporting Information). In general, neither ultrasound alone nor the +GVs condition used in our experiments were seen to trigger PI influx in primary neurons or CLU199 cells (Figure S3b,c, Supporting Information). Further, we did not observe obvious cytotoxicity or apoptosis in primary neurons following the treatments (Figure S3d,e, Supporting Information). Thus, we concluded that our GVs+US treatment could trigger calcium responses in cells with negligible loss of membrane integrity, which is consistent with stable cavitation hypothesis.

Given that we did not observe sonoporation to be the mechanism of calcium dynamics, we were interested in broadly identifying what mechanisms may have been involved in the cellular response to US+GVs. Mechanosensitive ion channels are a well‐established way in which cells transduce mechanical forces to cellular signaling. To assess their potential contribution, we used Ruthenium Red (RR, 20 µm), a blocker for a range of mechanosensitive ion channels,^[^ ^44^ ^]^ to see if the response to GVs+US would be altered. Calcium responses to ultrasound pulses were found to be significant, but not entirely, suppressed in the presence of RR, and the responses recovered when it was washed away (Figure 3b,c). We observed a similar suppression of Ca^2+^ influx in the presence of RR in CLU199 cells (Figure S2a–c, Supporting Information). We then tried to identify the source of Ca^2+^ responses by treating cells with GVs+US in EGTA‐chelated medium, or pretreating cells with Thapsigargin (TG, 3 µm) to deplete intracellular calcium stores.^[^ ^45^, ^46^ ^]^ Compared to normal conditions, cells in Ca^2+^‐free medium showed much reduced calcium influx (≈75% reduction), but the reduction in TG‐treated cells was much lesser (≈25–30% reduction) (Figure 3d,e and Figure S2a–c, Supporting Information). We thus found that while intracellular Ca^2+^ release played some role in the GVs+US response, calcium influx from the external medium had a much larger contribution to the observed outcomes. Further, when primary neurons were made to heterologously express the mechanosensitive ion channel MscL‐EYFP, or EYFP alone, the MscL‐EYFP+GVs showed a Ca^2+^ response to ultrasound several times stronger than that of EYFP+GVs (Figure 3f,g). Altogether, with no significant sonoporation being observed, the cellular response being significantly depressed when treated with RR or in Ca^2+^‐free medium but not in TG‐treated cells, and much greater response to US+GVs in cells with artificially‐enhanced mechanosensitivity, we inferred that activation of mechanosensitive ion channels was an important mechanism of GV‐mediated ultrasound stimulation.

### 2.4 GVs Enable Low‐Intensity Ultrasound to Activate Neurons in a Deep‐Seated Brain Region In Vivo

**Figure 4:**
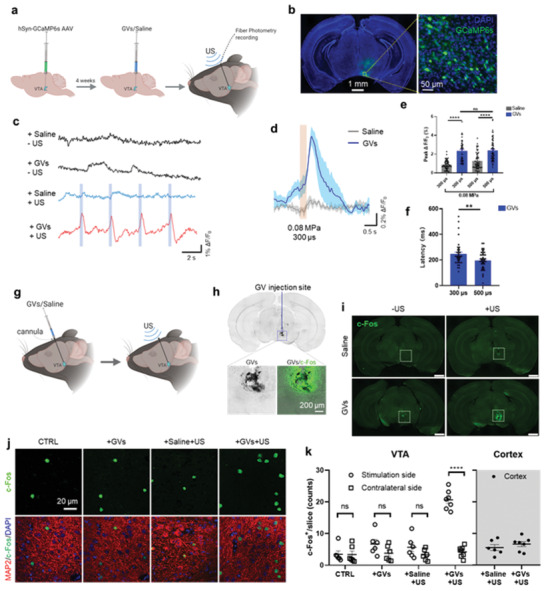
GVs enable efficient and non‐invasive ultrasound stimulation of a deep brain region in vivo. a) Schematic showing the hSyn‐GCaMP6s virus delivery, GVs or saline injection, fiber implantation and ultrasound stimulation in the mouse brain. b) Confocal images of hSyn‐GCaMP6s expression in the mouse VTA. c) Representative GCaMP6s fluorescence traces in the VTA of the anaesthetized mice in the presence of saline or GVs, before and after US (0.08 MPa peak pressure, 300 µs pulse width, 1 kHz PRF, 3 s burst interval). Light blue rectangle bars indicate ultrasound pulses. d) Averaged GCaMP6s fluorescence traces in the VTA of the anesthetized saline mice and GVs mice in response to ultrasound stimulation. *n* = 5 for both groups. e) Average peak Ca^2+^ activity in saline mice and GVs mice respond to different parameter ultrasound stimulation (300 or 500 µs pulse width, 0.08 MPa, 1 kHz PRF, 3 s burst interval). *n* = 5 mice in saline group, *n* = 5 mice in GVs group. ***P < 0.001, unpaired 2‐tailed *t*‐tests. Data are shown as mean ± SD. f) The latency between ultrasonic stimulation of specified pulse widths (300 or 500 µs pulse width) and detection of an above‐threshold response. *n* = 5 mice in saline group, *n* = 5 mice in GVs group. **P < 0.01, unpaired 2‐tailed *t*‐tests. Data are shown as mean ± SD. g) Schematic illustration of our GVs/saline injection and ultrasound stimulation plan. Briefly, mice at 8 weeks were embedded with cannulas in their VTA, and 1 week later, they were treated with pulsed US for 40 min (0.14 MPa peak pressure, 100 µs pulse width, 1 kHz PRF, 10 s burst interval). The mice were sacrificed after an interval of 90 min, and their brains were imaged for DAPI, MAP2, and c‐Fos expression. h) Location of GV injection in mouse brain overlaid on c‐Fos expression in the VTA of a mouse treated with GVs+US. i) Low‐magnification image of mice brains expressing c‐Fos, showing the pattern of c‐Fos expression in mice brains treated as indicated. VTA region with GVs injected is indicated by the white dotted square. Scale bars represent 1 mm. j) Representative images of mouse VTA treated with or without ultrasound/GVs, stained for c‐Fos expression. k) Counts of nuclear c‐Fos in 200 × 200 µm area per slice imaged. The bar chart represents means ± SD of c‐Fos+ cells per stained slice. Mice number, *n* for +US+GVs groups = 7, and *n* = 6 for all other groups. ***p < 0.001, multiple unpaired two‐tailed *t*‐tests with Holm–Sidak correction. All significant differences are indicated in the graph.

We next evaluated GVs’ ability to enhance the neuromodulatory capability of ultrasound in vivo by applying transcranial ultrasound to mouse brains with GVs injected in a specific region. We chose the VTA, a region seated relatively deep in the brain to demonstrate the ability of the GVs+US scheme to selectively stimulate even a deep region of the brain with non‐invasive ultrasound.^[^ ^28^ ^]^ Neurons in the VTAs of mice were made to express GCaMP6s through viral transduction, and 4 weeks later, GVs or saline were injected into the VTA and ultrasound treatment performed (**Figure** **4**a,b). Cellular responses to the stimulation, in the form of Ca^2+^ dynamics, were recorded through fiber photometry. In order to elucidate the ability of GVs to actuate ultrasound energy locally, the acoustic pressure was kept low for these experiments. Instead, the pulse width was varied which has the effect of varying the ultrasound “ON” time per pulse, thus transmitting different amounts of energy. We found a repeated, rapid, and consistent increase in GCaMP6s fluorescence to ultrasound pulses of very low intensity (0.08 MPa, 300 µs pulse width, 300 ms pulse duration) in the presence of GVs, but not without (Figure 4c,d). We did not observe any repeated pattern of fluorescence changes prior to the US stimulation. The ultrasound parameters used did induce some Ca^2+^ response in the saline+US group (300 µs, peak Δ*F*/*F*_0_ = 0.8801 ± 0.0564%; 500 µs, peak Δ*F*/*F*_0_ = 1.276 ± 0.1085%), but the peak amplitude in the GVs+US condition was significantly higher at both pulse widths (300 µs, peak Δ*F*/*F*_0_ = 2.321 ± 0.1741%; 500 µs, peak Δ*F*/*F*_0_ = 2.386 ± 0.1387%) (Figure 4e). Latency was found to be 246.9 and 196.7 ms for 300 and 500 µs pulses, respectively, indicating that the responses were dependent on the amount of ultrasound energy applied (Figure 4f). These values are in line with those reported previously by Yoo et. al.^[^ ^47^ ^]^

We further assessed neuronal activation by looking at c‐Fos expression in the brain region targeted by our treatment and compared it to other regions to evaluate the spatial specificity of our scheme. Cannula was embedded, and 1 week later, saline or GVs were administered to the VTA through a cannula, and then the mice were exposed to transcranial ultrasound (0.14 MPa) (Figure 4g). We found that the spatial extent of neuronal activation (bright green dots) was largely co‐located with GVs (visible as dark area in the brightfield image) distribution in the VTA (Figure 4h,i). Neuronal activity was triggered by GVs+US in the ipsilateral VTA of mice, resulting in a significantly higher number of c‐Fos‐positive cells (20.6657 ± 1.1453), but not in the contralateral VTA regions (4.2143 ± 0.663), or in any regions in which either GVs or ultrasound were not used (Figure 4j,k). Crucially, we did not find an increase in c‐Fos expression in the cortices of mice treated with GVs+US compared to Saline+US, indicating that the activation was likely not due to widespread auditory effects. GVs were detectable within mouse brains for up to 10 days, with a significant reduction in signal after day 6 (Figure S4a, Supporting Information). In the week post‐injection, the body weights of mice injected with GVs did not differ significantly from those injected with saline (Figure S4b, Supporting Information). Thus, the GVs could enable low‐intensity, non‐invasively‐delivered ultrasound to specifically stimulate the mouse VTA, and could remain intact for several days within the brain without obvious detrimental effects.

## 3 Discussion

A non‐invasive neuromodulation technique with high spatiotemporal resolution holds great potential for studying neural circuits and treating neurological conditions. Here we have presented a GV‐mediated non‐genetic toolkit for precise neuronal activation. Using a 1.0 MHz plane transducer, we show that GVs can serve as localized acoustic actuators and amplifiers to decrease the threshold of ultrasound intensity for precise neurostimulation. Implementing ultrasound neuromodulation with high spatiotemporal resolution can be challenging, and involves trade‐offs between different concerns.^[^ ^48^ ^]^ Higher frequencies of ultrasound can be used to create smaller focal spots, but also have reduced tissue penetration capabilities, and vice versa for lower frequencies. The significant variation between skulls may also have unpredictable effects on the efficacy of transcranial ultrasound. One solution to this has been to use phased‐arrays, creating better focus through the skull by employing the phase aberration correction method.^[^ ^48^, ^49^ ^]^ Alternatively, the plane and low‐frequency ultrasound generated by a single element ultrasound transducer can achieve deep and precise stimulation with the help of GVs. Our findings provide a demonstration of one method to non‐invasively stimulate specific brain regions with LILFU. A neuromodulation technique that does not require genetic modification and uses safe nanoparticles could help ultrasound research progress significantly, and perhaps aid its clinical transition.

Using nanoparticles to convert an external energy source into localized effects is an increasingly‐common approach to neuromodulation. Piezoelectric nanoparticles have been developed which are able to efficiently convert ultrasound energy to localized electric stimulation.^[^ ^50^ ^]^ Optoacoustic nanoparticles have been used to convert light to acoustic waves, which can then directly activate individual neurons.^[^ ^51^ ^]^ In contrast, using ultrasound's ability to penetrate deep through tissue, sono‐optogenetics utilizes mechanoluminescent nanoparticles to convert sound into light to achieve local neuromodulation in deeper brain regions.^[^ ^52^ ^]^ Recently, Lea‐Banks et al. reported that nanodroplets loaded with drugs can be combined with ultrasound to achieve targeted neuromodulation.^[^ ^53^ ^]^ In the same vein, the present study, builds upon the status of nano‐sized gas vesicles as efficient ultrasound contrast agents^[^ ^54^ ^]^ to use them as actuators for reliable and reversible neuron stimulation, and to enable spatial targeting of a desired area of the brain.

The Ca^2+^ signaling enabled through GVs+US could be significantly suppressed by inhibiting mechanosensitive ion channels with RR or with Ca^2+^ free medium, but not as much in TG^+^ medium. Combined with the lack of PI signal within cells following GVs+US stimulation leads us to believe that membrane pore formation and internal Ca^2+^ release were not the chief mechanisms underlying Ca^2+^ influx. We were also able to increase the observed Ca^2+^ influx in neurons by expressing a heterologous mechanosensitive ion channel. These findings indicate that both extracellular and intracellular calcium response are elicited by GVs+US, but also that mechanosensitive ion channels played an important role in mediating the ion currents. Previous studies have demonstrated that ultrasound can activate mechanosensitive ion channels, such as TREK‐1/2,^[^ ^55^ ^]^ MscL,^[^ ^56^ ^]^ Piezo1,^[^ ^24^ ^]^ Mscl‐G22S,^[^ ^38^ ^]^ TRPA1,^[^ ^57^ ^]^ and this phenomenon has been used to enable sonogenetic ultrasound stimulation. Our GVs+US strategy somewhat parallels sonogenetics, using GVs as actuators of ultrasound to decrease the threshold of ultrasound‐induced neuronal activation with localized stimulation effects. However, this approach offers the potential to target cells even more specifically because the GVs’ protein shells are conducive to surface modification. Specific cell surface receptors could be targeted by attaching molecules (e.g., hyaluronic acid or folic acid) or antibodies to the GV shell, which could enable US stimulation with greater precision. Indeed, such an approach was used to specifically stimulate neurons in vivo through magnetothermal stimulation using magnetic nanoparticles.^[^ ^58^ ^]^ We envision that such an approach could help to further improve the targetability and efficacy of US neurostimulation.

While our in vivo scheme was as minimally‐invasive as possible, we still needed to perform some surgery to deliver the GVs into the brain. There are two possible ways to achieve non‐invasive delivery of GVs in targeted regions: 1) systematic delivery from the bloodstream; 2) genetically‐encoded expression. Surface‐modified GVs have been shown to be able to enter tumor vasculature^[^ ^59^ ^]^ and to penetrate the blood–brain barrier (BBB),^[^ ^60^, ^61^ ^]^ which could be one approach to minimize surgical invasion. Another possibility is to exploit the size difference between GVs and MBs, and use MBs to open the BBB, which would allow GVs to enter the targeted brain region.^[^ ^62^, ^63^, ^64^ ^]^ Alternatively, mammalian cells have been genetically engineered to express GVs as acoustic reporter genes (ARGs) to enable ultrasound imaging of gene expression.^[^ ^65^ ^]^ This approach has the advantage of being able to target specific cell types, easily achieved by placing the ARGs under different promoters in the viral design. Such application of ARGs could be a milestone development for ultrasound imaging, almost analogous to the role of green fluorescent protein in optical imaging. Combined with the GVs’ molecular imaging capability, we imagine that GVs+US could possibly be developed to have a more theranostic role in the brain in future.

## 4 Experimental Section

### Gas Vesicle Preparation

*Anabaena flos‐aquae* was cultured in sterile BG‐11 medium at 25 °C under fluorescent lighting with 14 h/10 h light/dark cycle. GVs were isolated by hypertonic lysis to release GVs by quickly adding sucrose solution to a final concentration of 25%. GVs were isolated by centrifugation at 400 x *g* for 3 h after lysis. To purify GVs, the solution was washed by the same centrifugation process three times and stored in PBS at 4 °C. The GVs’ concentration was measured by optical density at 500 nm (OD_500nm_) by a UV–visible spectrophotometer.^[^ ^31^ ^]^

### Passive Cavitation Detection

Acoustic spectroscopy on GV suspensions were performed in a custom‐built chamber, and the 1 MHz flat transducer and hydrophone (HGL‐0200, Onda) were perpendicularly aligned and immersed in a tank of deionized, degassed water (Figure S1c, Supporting Information). A rectangular agarose (3%) chamber of wall thickness 5 mm and cavity 15 × 15 mm was placed in the middle, with the center point 17.5 mm away from both the transducer and the hydrophone. 1 MHz sinusoidal trains of burst width 200 µs and burst interval 2 ms were generated by a function generator (AFG251, Tektronix), amplified by a radio frequency amplifier (A075, Electronics & Innovation Ltd.), to drive the emitting transducer, producing acoustic output with 0.28 MPa peak negative pressure. Signals received by the hydrophone were amplified (AH‐2010, Onda) and digitized (CSE1222, GaGe) before analysis. 20 sections of 200 µs digitized signal in 20 separate bursts were processed with fast Fourier transform using MATLAB and the resulting frequency spectra were averaged.

### Cell Culture

All cells were grown inside a standard humidified cell culture incubator at 37 °C with 5% CO_2_. CLU199 cells were routinely maintained in DMEM culture medium supplemented with 10% FBS and 1% Pen‐Strep (all from Gibco) and seeded on poly‐l‐lysine (PLL)‐coated glass coverslips as needed, allowed to grow overnight and used for experiments thereafter.

Neurons from rat embryos at embryonic day 18 were obtained as previously described.^[^ ^66^ ^]^ Briefly, cortices were dissected and treated with 0.25% trypsin for 15 min at 37 °C, followed by gentle mixing. The digestion was stopped with Neurobasal medium (Gibco) with 10% fetal bovine serum and 1% penicillin–streptomycin. The cells were resuspended in medium and gently mechanically triturated with a pipette, and then allowed to stand for 15 min. The resultant supernatant was discarded, and the cells were resuspended in the abovementioned medium and plated at 1 × 10^5^ cells cm^−2^ in 35 mm dishes with PLL‐coated (Gibco) coverslips or PLL‐coated glass‐bottomed confocal dishes. After 24 h, the medium was changed to Neurobasal + 2% B27 + 0.25% l‐Glutamine + 1% Penicillin‐Streptomycin (all from Gibco). Half the medium was replaced every 2–3 days. Cultured neurons were transducted with AAVs on day 7 and were used in experiments between DIV 10–12 (3–5 days post‐transduction). AAVs used in vitro were rAAV/9‐hSyn:MscL‐G22S‐F2A‐EYFP‐WPRE‐pA, expressing MscL‐G22S‐EYFP with a human synapsin promoter, and its vector control rAAV/9‐hSyn:EYFP‐WPRE‐pA (BrainVTA (Wuhan) Co. Ltd).

### Acoustic Field Characterization

A flat transducer with center frequency 1.0 MHz (A303S, Olympus) was employed in this study. Ultrasonic pulses were generated using a function generator (AFG251, Tektronix) and power amplifier (A075, Electronics & Innovation Ltd.). For ultrasound stimulation, the planar transducer with a diameter of 1.0 cm was fixed perpendicularly facing downward. Acoustic intensity profile was characterized by a hydrophone.

### In Vitro Ultrasound Stimulation

For the present study, a customized system which facilitated ultrasound stimulation and calcium imaging simultaneously was used (Figure 2a). Briefly, the ultrasound stimulation system was aligned with a calcium imaging system and the calcium responses of the stimulated neurons were monitored. Ultrasound was delivered through a waveguide filled with degassed water that was attached to the ultrasound transducer assembly. Cells were cultured on glass coverslips placed inside a culture dish, and GVs were added to the medium and gently mixed just before stimulation. Prior to cellular stimulation, the acoustic pressure and field produced by this setup were tested using a hydrophone, and was found that it provided a relative homogeneous ultrasound field in the central region (Figure S1d, Supporting Information). Each stimulus was composed of 300 tone burst pulses at a center frequency of 1.0 MHz, 10% duty cycle, pulse repetition frequency (PRF) of 1 kHz, at low acoustic intensities (0.03–0.20 MPa). These parameters amounted to ultrasound being delivered in very short bursts, minimize thermal effects. For experiments not involving real‐time imaging, cells were treated inside a standard cell culture incubator, as described in a previous study.^[^ ^38^ ^]^ Parameters for stimulation in these experiments were the same as mentioned above, with a slightly different range of acoustic pressures and a treatment time of 15 min.

### Calcium Imaging

Culture medium was replaced with Fluo‐4 AM (5 µm) or X‐Rhod‐1 AM (10 µm) (both from Invitrogen) working solution in Ca^2+^ solution (pH 7.4), and the cells were incubated at 37 °C in the dark for 30 min. Subsequently, a fresh Ca^2+^ solution was used to flush away excess dye before ultrasound stimulation. In mechanistic studies, several different media were used. To remove extracellular Ca^2+^, the coverslip was placed Ca^2+^ free solution with 0.5 mm EGTA to ensure that residual Ca^2+^ was completely chelated. To monitor concurrent cell membrane sonoporation during Ca^2+^ response measurement, the coverslip was perfused with PI solution (100 µg mL^−1^ in Ca^2+^ solution, Invitrogen). RR solution (20 µm RR in Ca^2+^ solution, Tocris Bioscience) into the culture medium to evaluate the effect of mechanosensitive ion channels on US+GVs‐elicited Ca^2+^ response. 0.20 mm Triton X‐100 was added to cells as a positive control of membrane permeability.

Calcium imaging was done with a modified inverted epifluorescence microscope. The excitation light was generated by a dual‐color LED, filtered and delivered to the sample to illuminate the calcium sensor. To minimize phototoxicity, the LEDs were triggered at 1 Hz and synchronized with sCMOS time‐lapse imaging. Coverslips with dye‐loaded or GCaMP6s‐expressing cells were placed above the objective, and GVs were distributed into the media directly before ultrasound stimulation. A camera was used to record the intracellular Fluo‐4 AM/X‐Rhod‐1 AM images with defined time intervals from a function generator at excitation wavelengths of 494 nm for Fluo‐4 AM or 580 nm for X‐Rhod‐1 AM. A brightfield image was taken to register the morphology of the cell immediately before and after the GVs mediated ultrasound stimulation. Software was used to communicate and coordinate the operation sequence between the microscope and monochromator.

### MTT Assay

MTT assays was used to evaluate cytotoxicity at different concentration of GVs mediated ultrasound stimulation in the treated CLU199 cells. Cells were treated with GVs alone, or US+GVs in 96‐well or 24‐well plates. After the indicated treatments and incubations, cells were incubated with 0.5 mg mL^−1^ MTT in the medium for 3–4 h at 37 °C, solubilized with DMSO and 15‐min shaking, and the solutions’ absorbance at 570 nm was read using an LEDTect 96 microplate reader.

### Cellular GVs Uptake Monitoring

GVs labeled with NHS‐Fluorescein (GVs‐Fluor) (Thermo Scientific, 46 409) were incubated with neurons at 37 °C for 1, 2, 6, 12, 24, and 48 h. Cells were washed with PBS to remove free GVs, and cellular uptake of GVs was observed by photographing green fluorescence inside the cells at these time points (excitation/emission wavelength: 494/518 nm). Cell condition was also recorded by taking phase contrast images.

### Immunocytochemical Staining

Cells were treated, allowed to incubate for 90 min and fixed using 4% paraformaldehyde + PBS and permeabilized using 0.1% Triton X‐100 + PBS, and washes were done with 1× PBS or 1× PBS+Tween‐20 (PBST) (after permeabilization). Cells were blocked with 2% BSA + 0.3 m glycine + PBST, and incubated overnight with primary antibodies in 2% BSA + PBST. The next day, cells were washed and incubated with secondary antibodies in 2% BSA + PBST, then washed and mounted with Fluoroshield mounting medium with DAPI (Abcam). Stained cells were imaged on a Leica TCS SP8 confocal microscope. All steps from secondary antibody incubation onward were performed in the dark.

Primary antibodies used were c‐Fos (Cell Signaling #2250) at a dilution of 1:3000, and MAP2 (PA1‐10005, Invitrogen) at a dilution of 1:2500. Secondary antibodies, used at a dilution of 1:1000, were Goat anti‐Rabbit IgG Alexa Fluor Plus 488 (#A32731), Goat anti‐Chicken IgY Alexa Fluor Plus 555(#A32932) or Goat anti‐Rabbit IgG Alexa Fluor Plus 555 (#A32732), all from Invitrogen.

The number of c‐Fos^+^ cells in primary neurons was determined by counting the number of neuronal nuclei showing c‐Fos expression 90 min after stimulation. For non‐transducted cells, nuclear c‐Fos was counted in cells staining positive for MAP2. For transducted cells, nuclear c‐Fos was counted in cells showing EYFP expression. The percentage of cells showing c‐Fos among the cells identified was then calculated per experiment. The number of EYFP^+^ and EYFP^−^ cells with nuclear c‐Fos expression in MscL‐transducted dishes were also calculated. Each experiment had a minimum of 10 photographed FOVs and minimum of 50 total cells counted per condition.

### Animal Care

Male C57BL/6 were purchased from Jackson Laboratories, and housed under standard housing conditions, with food and water available ad libitum. Animals from the abovementioned groups were assigned randomly to treatment groups. All animal experiments were approved by the Animal Subjects Ethics Sub‐Committee (ASESC) of the Hong Kong Polytechnic University, and were performed in compliance with the guidelines of the Department of Health—Animals (Control of Experiments) of the Hong Kong S.A.R. government.

### Stereotactic Injection of Virus

C57BL/6 mice at 8 weeks were anesthetized with Ketamine and Xylazine (100 and 10 mg kg^−1^ respectively) via an intraperitoneal injection. The anaesthetized mice were positioned in the stereotaxic apparatus, and ointment was applied on the eyes. Skin incisions were then performed to expose the skull. 0.5 µL AAV9‐hSyn:GCaMP6s was injected into the VTA at 0.05 µL min^−1^, followed by a 10‐min pause. The pipette was then slowly withdrawn. After finishing the injection, skin tissue was sutured and disinfected, and mice were allowed to recover on a heating pad. The coordinates used for the VTA region were AP: −2.90 mm, ML: −0.50 mm, DV: −4.50 mm. Mice were returned to their housing areas. GVs and saline were injected around 4 weeks after virus expression for fiber photometry.

### Cannula Implantation and GVs Injection

8 week‐old C57BL/6 mice were anesthetized with Ketamine and Xylazine (100 and 10 mg kg^−1^, respectively) followed by shaving the skin above chosen VTA region. Using a stereotaxic apparatus, a hole was drilled to embed cannulas. The coordinates used for cannulas were AP: −2.90 mm, ML: −0.50 mm, DV: −4.50 mm, which were then fixed in place with dental silicate cement. The puncture site was then disinfected and sutured, and the mice were returned to their housing areas. After 1 week of recovery, injection sites variously received 1.0 µL of GVs (8.0 nm in saline) or at 0.1 µL min^−1^, followed by a 10‐min pause. The body weights of mice were monitored as a general indicator of health. In some mice, ICG‐labeled GVs^[^ ^59^ ^]^ were used, and the lifetime of GVs’ survival in vivo was monitored at 2, 6, 8, and 10 days using the IVIS imaging system (PerkinElmer Inc.) (excitation/emission wavelength: 774/805 nm).

### In Vivo Ultrasound Stimulation

Shortly after delivering the GVs, a 1.0 MHz transducer was coupled to the head with ultrasound gel. Mice were treated with ultrasound for 40 min, after which they were maintained in an anesthetized state for 90 min.

### Immunohistochemical Staining

Anesthetized mice were perfused transcardially with PBS and 4% paraformaldehyde (PFA) (cat. no. P1110, Solarbio) 90 min after completion of ultrasound treatment. Isolated brains were fixed in 4% PFA overnight, and 40 µm‐thick coronal slices were cut on a vibratome. Targeted brain slices were collected then blocked for 90 min in blocking medium (0.3% TritonX‐100 and 10% normal goat serum with 1% BSA) followed by overnight incubation with primary antibody at 4 °C. After two washes of 15 min with PBS, brain slices were incubated with secondary antibody for 2 h at room temperature. Following three more washes with PBS, the slices were mounted on glass slides using drops of Prolong Diamond Antifade Mountant with DAPI. Primary antibodies used were MAP2 (PA1‐10005, Invitrogen, diluted 1:1000) and c‐Fos (#2250, Cell Signaling Technology, diluted 1:500). Secondary antibodies used at a dilution of 1:1000 were goat anti‐chicken IgY (H+L) Alexa Fluor 555 (A‐21103, Invitrogen) and goat‐anti‐rabbit IgG (H+L) Alexa Fluor 488 (A‐21428, Invitrogen). The number of nuclei showing c‐Fos signals (green) were counted using ImageJ, and the number of c‐Fos^+^ cells per 200 × 200 µm slice were calculated. The counting of c‐Fos^+^ cells was single‐blinded, performed by an experimenter who did not know the groups beforehand. All brain slices were imaged using a confocal microscope (TCS SP8, Leica) in the ULS facilities in The Hong Kong Polytechnic University.

### Fiber Photometry Recording

Mice were anesthetized with isoflurane (0.5–2.5% in O_2_) with an anesthesia machine. An ointment was applied to the eyes to prevent drying. Anaesthetized mice were placed in a stereotaxic apparatus. A small section of skull was removed (0.5 mm × 0.5 mm) after removing the hair and skin. A syringe was placed directly above the skull and filled with GVs (8.0 nm) or saline solution. 1.0 µL GVs or saline at 0.1 µL min^−1^ was injected into the VTA. The syringe was then slowly withdrawn and a 10 min pause was allowed after completing the injection. A fiber optic cannula was then implanted into the VTA. The cannula was fixed to the skull with glue and dental cement and allowed to set for 20 min. Mice were then moved back to their original housing for up to 1 week.

Following implantation and recovery, mice were anesthetized with isoflurane (1.0–2.5% in O_2_). Ultrasound gel was applied on a shaved head, to promote acoustic coupling. A 1.0 MHz transducer embedded with a water tube wave‐guide was installed above the skull. Mice were stimulated with two trials of ultrasound stimulation (0.08 MPa with 300 or 500 µs pulse width, 1 kHz PRF, 300 ms stimulation duration). Each trial was 5–18 ultrasound stimuli, with 3 s interval between each pulse. Mice were allowed to rest for 45 s between trials. GCaMP6s fluorescence was captured with a fiber photometry system (Thinker Tech Nanjing BioScience Inc.). The excitation and receiving wavelength for fiber photometry were 470 nm with 30 nm bandwidth and 510 nm with 25 nm bandwidth, respectively. Data was collected at 100 Hz and analyzed using a customized MATLAB script.

### Statistical Analysis

GraphPad Prism was the software used for statistical analyses of all data and to prepare the graphs. Two‐tailed unpaired *t*‐tests, or one‐ or two‐way ANOVA were performed to determine statistical significance, with post‐hoc tests or corrections applied where appropriate. *P* values below 0.05 were considered significant. Figure legends indicate the specific statistical test applied for each panel. Data are presented as mean ± S.E.M. or mean ± S.D. as indicated.

## Conflict of Interest

The authors declare no conflict of interest.

## Author Contributions

X.H., Z.Q., and Q.X. contributed equally to this work. X.H., Z.Q., and L.S. contributed to the conceptualization; X.H., Z.Q., S.K., Q.X. J.G., and L.S. contributed to the methodology; X.H., S.K., Q.X., J.J., K.F.W., T.Z., and M.Y. contributed to the investigation; X.H., Z.Q., S.K., J.J., and Q.X. contributed to the data analysis; X.H., Z.Q., S.K., and L.S. contributed to the manuscript preparation; L.S. contributed to the funding acquisition.

## Supporting information

Supporting Information

Supporting Information

Supporting Information

## Data Availability

Research data are not shared.
