# Supplementary material for: Precise Ultrasound Neuromodulation in a Deep Brain Region Using Nano Gas Vesicles as Actuators
Source: Adv Sci (Weinh). 2021 Sep 21;8(21):2101934. doi: 10.1002/advs.202101934 (PMC8564444; doi:10.1002/advs.202101934)
Supplement: Supplementary file 1 — Supporting Information [file ADVS-8-2101934-s002.pdf]

© 2021 Wiley-VCH GmbH

ADVANCED SCIENCE
Open Access

Supporting Information

for *Adv. Sci.,* DOI: 10.1002/advs.202101934

Precise Ultrasound Neuromodulation in a Deep Brain Region Using Nano Gas Vesicles as Actuators

*Xuandi Hou, Zhihai Qiu, Quanxiang Xian, Shashwati Kala, Jianing Jing, Kin Fung Wong, Jiejun Zhu, Jinghui Guo, Ting Zhu, Minyi Yang, and Lei Sun**

Supporting information

Supplementary Methods

Supplementary Figures. 1 - 4

Legends for Supplementary Videos 1 - 2

Supplementary Videos 1 - 2

Supplementary Methods

Western Blot

Primary neurons were treated with GVs and US in various combinations, and the treatments' apoptotic effects were evaluated by a WB of caspase-3. Cells were treated inside an incubator for 15 minutes, allowed to incubate overnight, and protein was collected using RIPA buffer supplemented with 1X Halt Protease and Phosphatase Inhibitor Cocktail (Thermo Scientific). Cells were run on a 4 - 20% Tris-Glycine SDS-PAGE gel, transferred to activated PVDF membrane (Millipore), and incubated overnight with caspase-3 primary antibody (Cell Signaling #9662) diluted 1:1,000 or α-tubulin primary antibody (Proteintech # 66031-1-Ig) diluted 1:2,500 in 5% milk + TBST. Membranes were washed with TBST, and incubated at room temperature with Goat anti-Rabbit IgG (H+L) superclonal secondary (Invitrogen #A27022) or Rabbit anti-Mouse IgG (H+L) superclonal secondary antibody (Invitrogen #A27033), diluted at 1:20,000 in 5% milk + TBST. Signals were developed using SuperSignal West Pico PLUS Chemiluminescent Substrate and visualized on a ChemiDoc MP imaging system (Bio-Rad). Proteins were quantified using image densitometry and normalized to the α-tubulin expression levels with ImageJ.

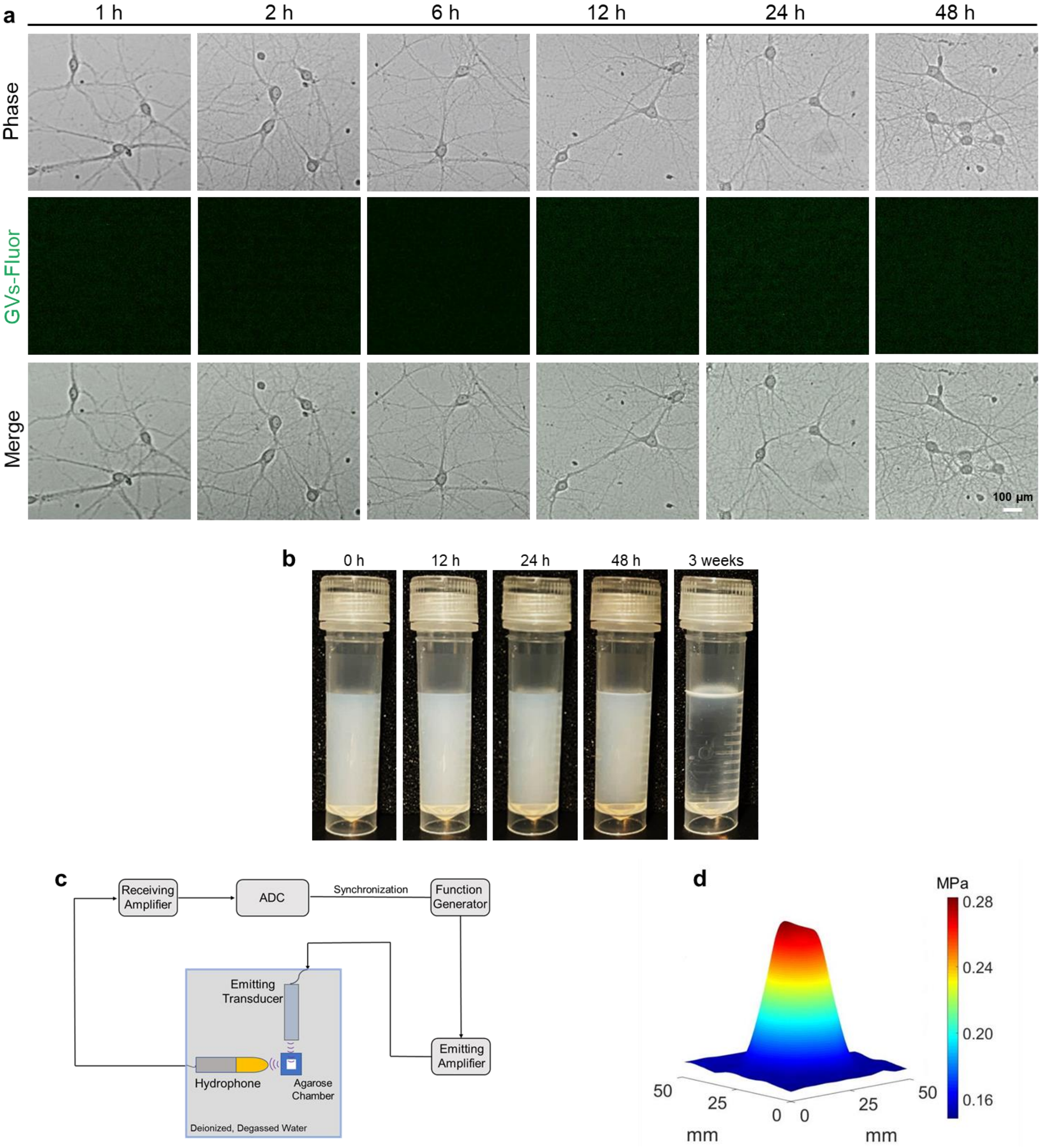

Supplementary Figure 1. Characterization of GVs' properties culture medium and in solution, and characterization of GVs+US setup

a. Fluorescein-labeled GVs were incubated with neurons for indicated periods of time. Cellular uptake of GVs and cell condition were monitored through fluorescent and phase contrast microscopy respectively. Scale bar represents 100 µm. **b.** The buoyant properties of GVs suspended in PBS were evaluated by photographing them in a tube at the indicated time points. **c.** The *in vitro* passive cavitation detection system used to measure the backscattered acoustic signals of cavitation in response to GVs+US stimulation. **d.** Acoustic field characterization of our stimulation setup, with a spatial PNP of 0.28 MPa.

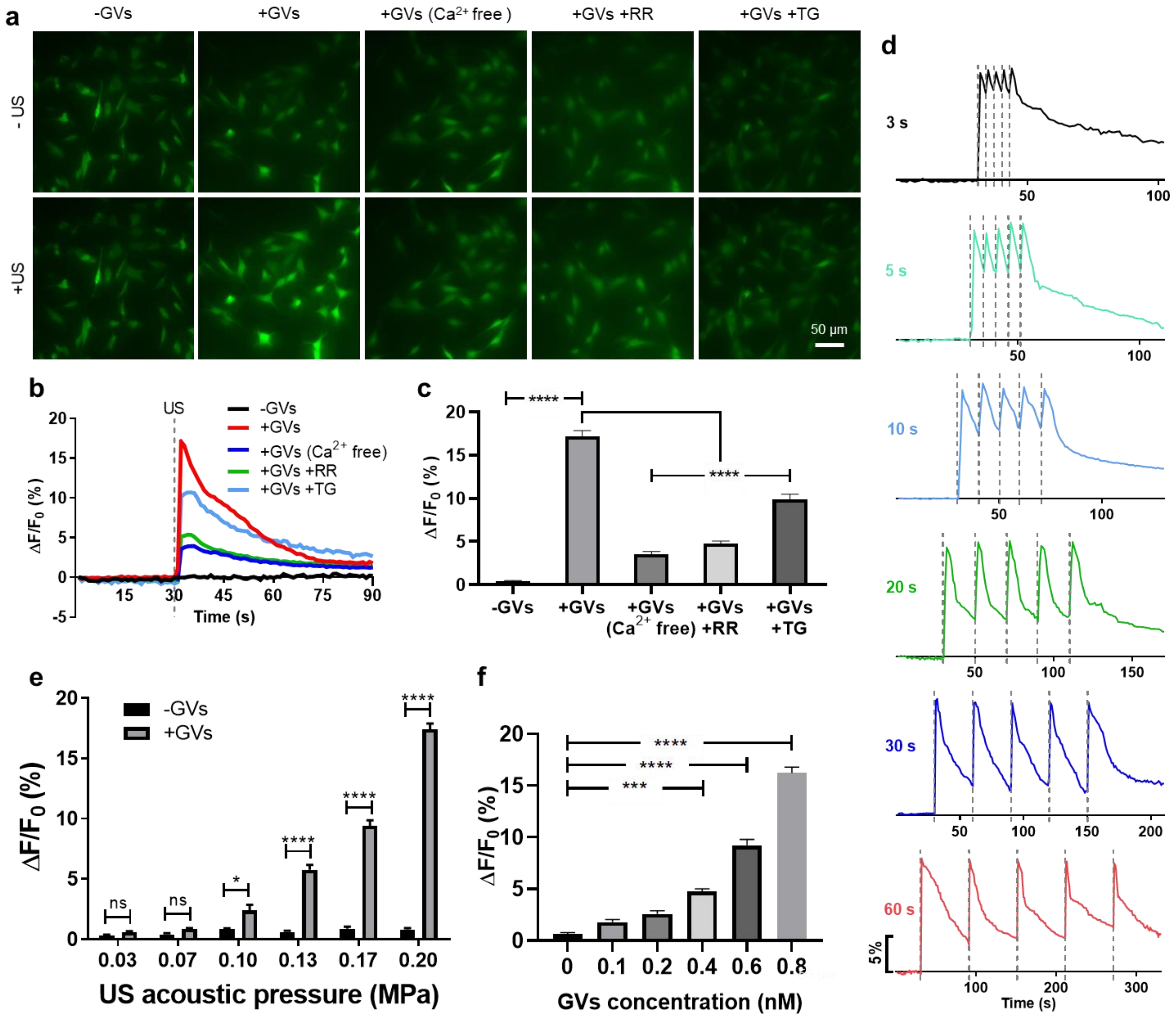

Supplementary Figure 2. Calcium imaging of ultrasound + GVs stimulation performed in the neuronal cell line, CLU199.

a. Representative images of the typical Ca^{2+} response seen in CLU199 cells before and after 0.20 MPa ultrasound stimulation with or without GVs, in calcium-free medium, with the broad-spectrum mechanosensitive ion channel blocker ruthenium red (RR) or with the internal calcium chelator Thapsigargin (TG). **b.** Time-course of the imaging results depicted in **a**. **c.** Quantification of the fluorescence intensity changes shown in **a** and **b**. Bars represent mean ± SEM of 3 independent experiments. ****p < 0.0001 compared only to the +GVs condition, unpaired one-way ANOVA with post-hoc Dunnett test. **d.** Time-resolved Ca^{2+} responses of CLU199 cells stimulated by 5 ultrasound pulses at varying intervals. **e.** Ca^{2+} response of cells to varying ultrasound intensities, 0.8 nM GV. Bars represent the mean ± SEM of 3 independent experiments. *p < 0.05, ****p < 0.0001; two-way ANOVA with Sidak correction. **f.** Ca^{2+} response of cells to varying GVs concentrations, 0.20 MPa ultrasound. Bars represent the mean ± SEM of 3 independent experiments. *p < 0.05, ****p < 0.0001 compared only to the 0 nM GVs condition, unpaired one-way ANOVA with Dunnett correction.

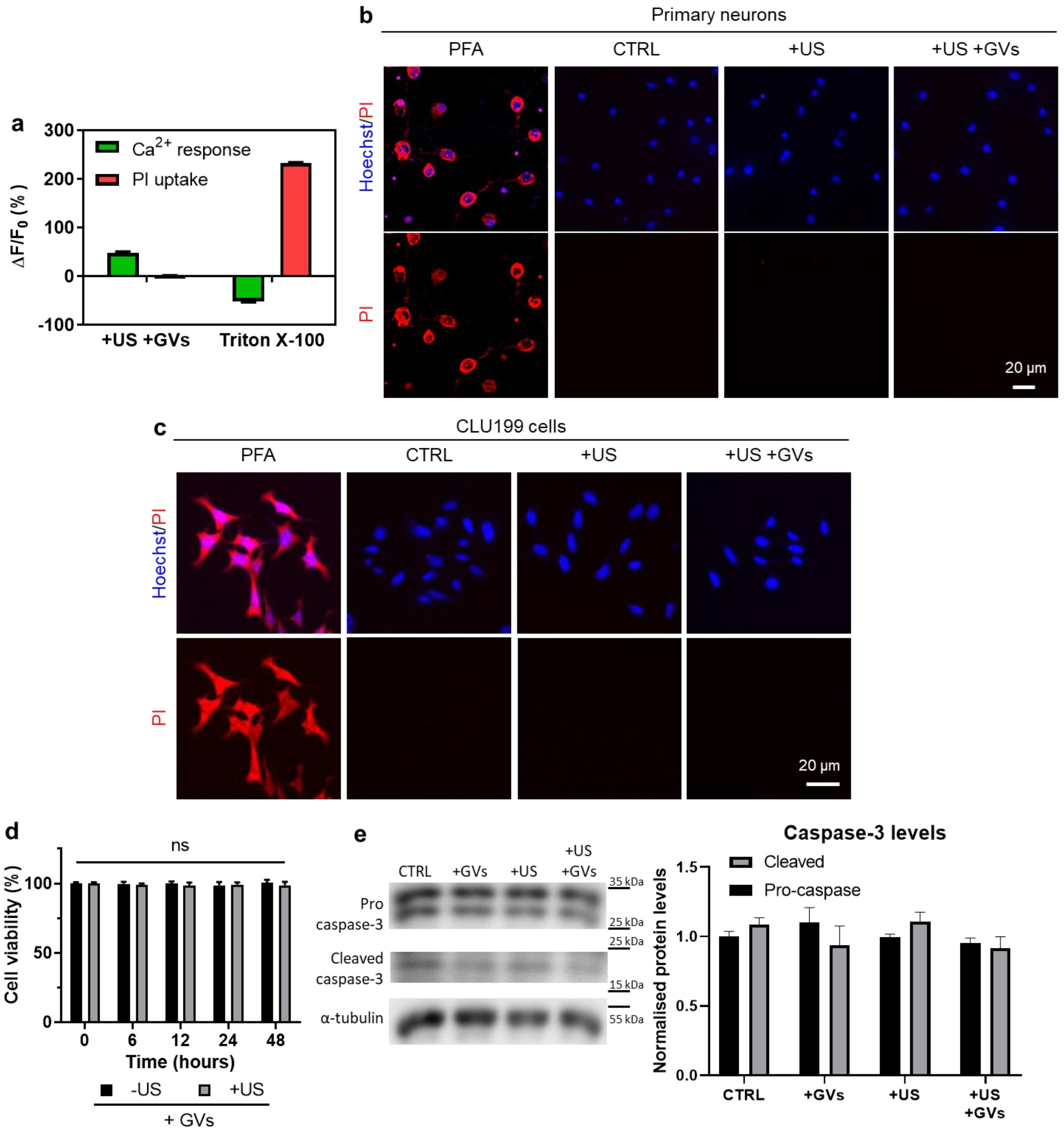

Supplementary Figure 3. Evidence for non-cytotoxicity of the GVs+US scheme.
a. Quantified fluorescence changes of neuronal Ca^{2+} response and propidium iodide (PI) uptake of the representative images shown in Figure 3a. Bars represent mean ± SD from 3 independent experiments. **b.** Intracellular uptake of PI by primary neurons when untreated, treated with ultrasound-alone or with ultrasound + GVs (0.20 MPa, 10 seconds burst interval, 10% duty cycle, 0.8 nM GVs). Primary neurons treated with 4% paraformaldehyde (PFA) are shown here as a positive control for membrane permeation and PI staining. **c.** Intracellular uptake of PI by CLU199 cells. All treatment conditions were the same as in **b. d.** Cell viability following US+GVs treatments. CLU199 cells were treated with either GVs alone or US+GVs for 15 minutes, and their cell viability at various times post-treatment was determined using an MTT assay. Bars represent the mean ± SEM of 3 independent experiments. No significant differences found, multiple two-tailed *t*-tests with Holm-Sidak correction. **e.** Caspase-3 levels in primary neurons following various treatments. Primary neurons were exposed to either GVs, ultrasound or ultrasound + GVs for 15 minutes, proteins were collected after overnight incubation and a WB was performed to observe levels of pro- and cleaved caspase-3. Only upper bands were quantified for pro caspase-3. Bars represent the mean ± SEM of 3 independent experiments. No significant differences found, two-way ANOVA with post-hoc Tukey test.

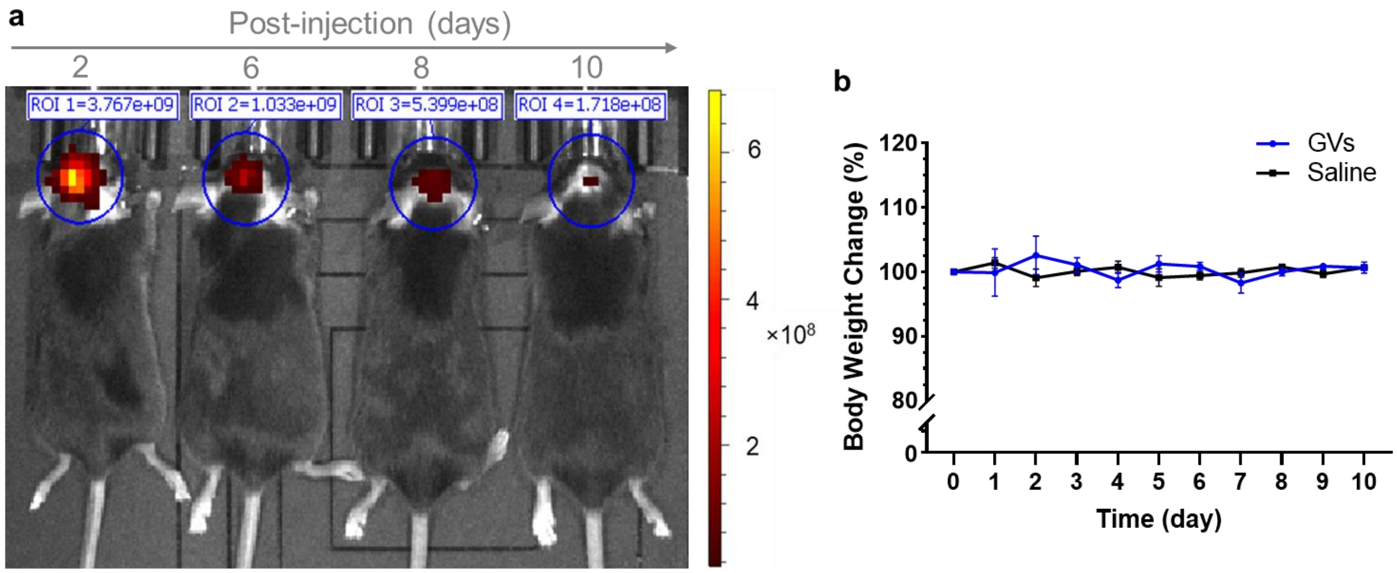

Supplementary Figure 4. Monitoring of mice with GVs injection in the brain.

a. *In vivo* NIR fluorescent imaging of ICG-labeled GVs injected mouse brains at different days (2, 6, 8, and 10 days). **b.** Body weight measurement of mice during the 10-day evaluation period. Data represent the mean ± SD from n = 3 in both groups

Figure Legends for Supplementary Videos 1 – 2

Video 1. Primary neurons show rapid and reversible calcium influx in response to each ultrasound pulse (0.20 MPa, 8 pulses) in the presence of GVs (0.8 nM).

Video 2. GV-mediated ultrasound stimulation (0.20 MPa) triggers calcium influx into primary neurons, without allowing PI to enter cells due to membrane poration.
